# Comparison of Two Different Cut-Off Values of Scoring System for Diagnosis of Hirschsprung-Associated Enterocolitis After Transanal Endorectal Pull-Through

**DOI:** 10.3389/fped.2021.705663

**Published:** 2021-08-16

**Authors:** Gunadi, Raedi Ardlo Luzman, Sagita Mega Sekar Kencana, Bhagas Dwi Arthana, Fauzan Ahmad, Ganjar Sulaksmono, Agitha Swandaru Rastaputra, Golda Puspa Arini, Ririd Tri Pitaka, Andi Dwihantoro, Akhmad Makhmudi

**Affiliations:** Pediatric Surgery Division, Department of Surgery, Faculty of Medicine, Public Health and Nursing, Universitas Gadjah Mada/Dr. Sardjito Hospital, Yogyakarta, Indonesia

**Keywords:** HAEC following pull-through, developing country, HAEC scoring system, transanal endorectal pull through, Hirschsprung disease, children

## Abstract

**Background:** Hirschsprung-associated enterocolitis (HAEC) is a major contributor in the mortality of Hirschsprung disease (HSCR) patients that can occur both preoperatively and post-operatively. Several cut-off values of HAEC score have been used, i.e., ≥10 and ≥4. Here, we compared the HAEC frequency after transanal endorectal pull-through (TEPT) using two cut-offs of scoring system and associated them with the risk factors.

**Methods:** Cross-sectional analysis was conducted using medical records of HSCR patients who were aged ≤18 years old and underwent TEPT at our institution, Indonesia between 2009 and 2016. HAEC was determined using the scoring system with cut-off values of ≥10 and ≥4.

**Results:** Seventy subjects were used in the final analysis, consisting of 44 males and 26 females. There was a significant difference in one HAEC finding between the ≥10 and ≥4 cut-off groups; diarrhea with explosive stools (*p* = 0.002). The HAEC frequency was 5/70 (7.1%) and 49/70 (70%) patients using cut-off values of ≥10 and ≥4 (*p* < 0.0001), respectively. We found that patients with anemia (i.e., iron deficiency anemia) had a higher risk of HAEC after TEPT than patients with normal hemoglobin level with OR of 3.77 (95% CI = 1.28–11.1; *p* = 0.027), while no associations were found between other variables, including sex, age at diagnosis, age at definitive therapy, albumin level, and nutritional status and HAEC following TEPT (*p* = 0.87, 0.15, 0.33, 0.26, and 0.60, respectively). Also, no associations were observed between maternal education level, mother's age at pregnancy and gestational age and HAEC after definitive surgery (*p* = 0.10, 0.46, and 0.86, respectively).

**Conclusions:** This report is the first study comparing two different cut-off values of scoring system to evaluate the HAEC frequency after TEPT and results suggest further using cut-off of ≥4 to expand the diagnosis of HAEC. Moreover, we also show for the first time that hemoglobin level is a strong risk factor for the HAEC development after TEPT.

## Introduction

Hirschsprung-associated enterocolitis (HAEC) is a major contributor of mortality for patients with Hirschsprung disease (HSCR) (1, 2). It can occur both preoperatively and post-operatively (3, 4).

The objective of surgical treatment for HSCR is to remove the aganglionic colon and to anastomose the ganglionic colon above the dentate line (1, 5). Transanal endorectal pull-through (TEPT) is the most current popular procedure in the surgical correction of HSCR since it is less invasive and has many advantages compared with the transabdominal approaches (5–9).

Various risk factors of HAEC after TEPT have been reported, such as sex, age at HSCR diagnosis and pull-through performed, however, they are still limited (10, 11). Moreover, several cut-off values of HAEC score have been used, i.e., ≥10 and ≥4 (12–15). Originally, the HAEC scoring criteria was determined using a Delphi-based consensus of experts that concluded that a score ≥10 was diagnostic of HAEC (12). Frykman et al. (16) performed a critical univariate and multivariate application on those scoring criteria, clustered some of the criteria and concluded that a score ≥4 optimized sensitivity and specificity. Therefore, they proposed a score ≥4 as diagnostic of HAEC (16). Several papers confirmed that the HAEC scoring system with cut-off of 4 increase the sensitivity, which is better for screening purposes (15, 17). Here, we compared the HAEC frequency after TEPT using two cut-offs of scoring system and associated them with the risk factors, including sex, age at HSCR diagnosis and TEPT performed, nutritional status, hemoglobin and albumin level, gestational age, mother's age during pregnancy, and maternal education level.

## Materials and Methods

### Subjects

We conducted a cross-sectional analysis using medical records of patients with HSCR who were aged ≤18 years old and underwent TEPT at our institution, Indonesia. Our institution is an academic and tertiary referral hospital for patients from two provinces, i.e., Special Region of Yogyakarta and southern part of Central Java, Indonesia. Medical records with incomplete data were excluded from analysis.

This study received prior approval from the Medical and Health Research Ethics Committee of the Faculty of Medicine, Public Health and Nursing, Universitas Gadjah Mada/Dr. Sardjito Hospital (KE/FK/786/EC/2015, KE/FK/713/EC/2015, and KE/FK/1356/EC/2015).

### TEPT

The surgical procedure was conducted based on previous reports (6, 7, 18). First, the anal mucosa was exposed by performing everting sutures throughout the anus. Subsequently, the mucosa about 0.5 cm above the dentate line was incised circumferentially. A submucosal dissection was conducted proximally about 1–2 cm, followed by full thickness of rectal wall conversion until the transition zone was determined. Frozen section was conducted to confirm the presence of the ganglion cells in the colon. Once the ganglion cells identified, as a minimum an additional 5 cm of colon was resected to make sure that the transition zone was removed along with the aganglionic colon. Final step was a colo-anal anastomosis.

### HAEC Scoring System

Diagnosis of HAEC was determined by scoring system, and included patient history, physical examination, radiologic, and laboratory findings (12–15). We used scores of ≥4 and ≥10 as cut-off values for diagnosis of HAEC (13–17).

### Risk Factors

We gathered data on sex, age at HSCR diagnosis, age at TEPT performed, weight and height before TEPT, preoperative albumin, and hemoglobin level.

We categorized hemoglobin and albumin level into anemia (i.e., iron deficiency anemia) and normal (19), and hypoalbuminemia and normal, respectively. The weight and height data were used to measure the nutritional status. Nutritional status of children younger than 5 years old was expressed as weight-for-age z scores (WAZ), while for children over 5 years old, it was expressed as BMI-for-age in relation to growth standards of the age and gender according to the WHO growth chart. We used WHO anthro-application to calculate the nutritional status. The scores gained were then classified as underweight-severely underweight (*z* < -2) and normal (+2> *z* >-2) (20, 21).

We also collected the gestational age, mother's age during pregnancy and maternal education level. For maternal education level, we categorized it into low (no education—elementary) and high (junior—high school—bachelor), while for gestational age and mother's age during pregnancy, we classified them into preterm and full-term and ≤ 35 and >35 years, respectively.

### Statistical Analysis

Sample size estimation was determined using an anticipated incidence of HAEC cut-off value of ≥10 and ≥4 of 30 and 70%, respectively, a confidence level of 95% and a power of 80%. The calculated minimum size required was 46 samples. Data were presented as number and percentage for categorical variables. Univariate analysis using Chi-squared or Fisher's Exact tests were performed for each categorical variable to evaluate the association between risk factors and HAEC incidence.

## Results

### Baseline Characteristics

We collected 75 medical records which fulfilled the inclusion criteria and excluded 5 medical records due to incomplete data. Thus, data from 70 subjects were used in the final analysis, consisting of 44 males and 26 females. Most of our patients were diagnosed (71.4%) and operated on (75.7%) at the post-neonatal period (Table 1). All patients had short-aganglionosis, non-syndromic, and underwent one-stage TEPT without any prior colostomy. The median of follow-up was 131 [interquartile range (IQR), 50.5–342.5] days after TEPT.

**Table 1:** Baseline characteristics of HSCR patients treated with TEPT procedure in our institution.

**Characteristics**	***N* (%)**
**Sex**	
Male	44 (62.9)
Female	26 (37.1)
**Age at HSCR diagnosis**	
Neonate (median = 20.5 [IQR, 14–26] days)	20 (28.6)
Post-neonate (median = 137 [IQR, 44–1078.25] days)	50 (71.4)
**Age at TEPT performed**	
Neonate (median = 21 [IQR, 15.75–26] days)	17 (24.3)
Post-neonate (median = 110.5 [IQR, 44.75–985.5] days)	53 (75.7)

*HSCR, Hirschsprung disease; TEPT, transanal endorectal pull-through; IQR, interquartile range*.

### Comparison of HAEC Frequency After TEPT Using Two Different Cut-Offs Scoring System

There was a significant difference in one HAEC finding between the ≥10 and ≥4 cut-off groups; diarrhea with explosive stools (*p* = 0.002, Table 2).

**Table 2:** HAEC findings in patients with HSCR after TEPT using different cut-off values.

**HAEC findings**	**Cut-off ≥10 (*n* = 5) *N* (%)**	**Cut-off ≥4 (*n* = 49) *N* (%)**	***p*-value**
**History**			
Diarrhea with explosive stool	5 (100)	12 (24.5)	0.002^*^
Diarrhea with foul-smelling stool	5 (100)	24 (49)	0.05
Diarrhea with bloody stool	1 (20)	2 (4.1)	0.26
History of enterocolitis	3 (60)	11 (22.4)	0.10
**Physical examination**			
Explosive discharge of gas and stool on rectal examination	4 (80)	19 (38.8)	0.15
Distended abdomen	5 (100)	46 (93.9)	1.0
Decreased peripheral perfusion	1 (20)	3 (6.1)	0.33
Lethargy	2 (40)	17 (34.7)	1.0
Fever	5 (100)	39 (79.6)	0.57
**Radiologic examination**			
Multiple air fluid levels	1 (20)	2 (4.1)	0.26
Dilated loops of bowel	1 (20)	13 (26.5)	1.0
Sawtooth appearance with irregular mucosal lining	1 (20)	1 (2)	0.18
Cut-off sign in rectosigmoid with absence of distal air	0	1 (2)	1.0
Pneumatosis	0	3 (6.1)	1.0
**Laboratory**			
Leukocytosis	2 (40)	15 (30.6)	1.0
Shift to left	0	15 (30.6)	0.31

* *p < 0.05 was considered significant; HAEC, Hirschsprung-associated enterocolitis; HSCR, Hirschsprung disease; TEPT, transanal endorectal pull-through*.

Next, we compared the HAEC incidence after TEPT using two cut-offs of scoring system. The HAEC frequency was 5/70 (7.1%) and 49/70 (70%) patients using cut-off of ≥10 and ≥4 (*p* < 0.0001), respectively.

### Association Between Risk Factors and HAEC After TEPT

We found that patients with anemia had a higher risk of HAEC after pull-through than patients with normal hemoglobin level with OR of 3.77 (95% CI = 1.28–11.1; *p* = 0.027) when we used cut-off value of ≥4 (Table 3). Apart from that, there were no associations between gender, albumin level, and nutritional status with HAEC incidence following TEPT procedure (*p* = 0.87, 0.26, and 0.60, respectively).

**Table 3:** Association between risk factors and HAEC after TEPT using cut-off value of ≥4.

**Variables**	**HAEC (*n* = 49)**	**Non-HAEC (*n* = 21)**	***p*-value**	**OR (95% CI)**
**Sex**				
Male	30	14	0.87	0.79 (0.27–2.31)
Female	19	7		Ref
**Age at diagnosis**				
Neonate	17	3	0.15	3.2 (0.82–12.38)
Post-neonate	32	18		Ref
**Age at TEPT performed**				
Neonate	14	3	0.33	2.4 (0.61–9.45)
Post-neonate	35	18		Ref
**Albumin level**				
Hypoalbuminemia (<3.5 g/dL)	15	3	0.26	2.65 (0.68–10.36)
Normal (≥3.5 g/dL)	34	18		
**Hemoglobin level**				
Anemia^ß^	32	7	0.027^*^	3.77 (1.28–11.1)
Normal	17	14		Ref
**Nutritional status**				
Under-nourished	26	9	0.60	1.51 (0.54–4.22)
Normal	23	12		Ref
**Mothers' age at childbirth (years)**				
≥35	7	2	0.46	1.58 (0.3–8.35)
<35	42	19		Ref
**Gestational age**				
Preterm	1	0	0.86	0.75 (0.03–19.21)
Full-term	48	21		Ref
**Maternal educational level**				
Low	13	2	0.10	3.43 (0.7–16.81)
High	36	19		Ref

* *p < 0.05 was considered significant; CI, confidence interval; HAEC, Hirschsprung-associated enterocolitis; HSCR, Hirschsprung disease; OR, odds ratio; Ref, reference; TEPT, transanal endorectal pull-through*.

ß *Iron deficiency anemia*.

We also observed no associations between maternal education level, mother's age at childbirth and gestational age and HAEC occurrence after TEPT (*p* = 0.10, 0.46, and 0.86, respectively).

However, none of the risk factors were associated with HAEC following pull-through when we used cut-off value of ≥10 (Table 4).

**Table 4:** Association between risk factors and HAEC after TEPT using cut-off value of ≥10.

**Variables**	**HAEC (n = 5)**	**Non-HAEC (n = 65)**	***p*-value**	**OR (95% CI)**
**Sex**				
Male	4	40	0.38	2.5 (0.26–23.66)
Female	1	25		Ref
**Age at diagnosis**				
Neonate	0	20	0.29	0.2 (0.01–3.82)
Post-neonate	5	45		Ref
**Age at TEPT performed**				
Neonate	0	17	0.36	0.25 (0.01–4.8)
Post-neonate	5	48		Ref
**Albumin level**				
Hypoalbuminemia (<3.5 g/dL)	1	17	0.62	0.71 (0.07–6.77)
Normal (≥3.5 g/dL)	4	48		Ref
**Hemoglobin level**				
Anemia^ß^	3	36	0.61	1.21 (0.19–7.72)
Normal	2	29		Ref
**Nutritional status**				
Under-nourished	3	32	0.50	1.55 (0.24–9.88)
Normal	2	33		Ref
**Mothers' age at childbirth (years)**				
≥35	0	9	0.69	0.54 (0.03–10.6)
<35	5	56		Ref
**Gestational age**				
Preterm	0	1	0.86	3.91 (0.14–107.8)
Full-term	5	64		Ref
**Maternal educational level**				
Low	1	14	0.71	0.91 (0.09–8.81)
High	4	51		Ref

*CI, confidence interval; HAEC, Hirschsprung-associated enterocolitis; HSCR, Hirschsprung disease; OR, odds ratio; Ref, reference; TEPT, transanal endorectal pull-through*.

ß *Iron deficiency anemia*.

## Discussion

### Comparison of HAEC Frequency After TEPT Using Two Different Cut-Offs Scoring System

HAEC has remained a serious complication for patients with HSCR despite the various advancements in diagnosis and therapy. Its pathogenesis and etiology continue to be enigmatic (22). Previous studies about definitive treatment and associated risk factors of HAEC have obtained inconsistent findings (8, 9, 13). Moreover, several cut-offs of scoring system for HAEC diagnosis have been proposed (12, 13, 15–17). Our study intended to compare the HAEC frequency after TEPT between two cut-offs scoring system and identify any associated risk factors. Here, we found that the HAEC frequency after TEPT procedure in our institution was 10-times higher when using cut-off value of ≥4 (~70%) than when using cut-off value of ≥10 (~7%). These findings support our previous study that cut-off value of ≥4 increases the possibility to diagnose HAEC, particularly the mild one (15). However, at least two novelties were noted in our current study: (1) we compared two cut-off values of HAEC scoring system in patients following transanal-approach pull-through [vs. transabdominal Soave and Duhamel pull-through (15)]; and (2) our study provided a new evidence supporting the use of cut-off value of ≥4 to increase the possibility to diagnose HAEC after TEPT from a developing country setting [vs. developed countries (16, 17)].

Frykman et al. suggested that the HAEC scoring system with cut-off of 4 [sensitivity 83.7%; specificity 98.6%, AUC 0.910 (0.85–0.97)] should be used rather than 10 [sensitivity 41.9%; specificity 100%, AUC 0.744 (0.669–0.820)] to prevent under-diagnosing patients with HAEC (16). Thus, lowering the cut-off score could double the incidence of HAEC. Here, we got the average Delphi score of 6.59 ± 2.081, which means many patients would be underdiagnosed if we had used 10 as our cut-off point.

### Association Between Risk Factors and HAEC After TEPT

Interestingly, the hemoglobin level was significantly associated with HAEC incidence after TEPT (*p* = 0.027, Table 3). Anemia might induce dysbiosis in infant intestinal lumen which may contribute to the development of HAEC together with alterations in the intestinal barrier and impaired GI mucosal immunity (22, 23). Patients might have better nutrition and oxygen transport during an operation in a non-anemic condition which would eventually improve the surgery outcome. To the best of our knowledge, our study is the first report showing the effect of iron deficiency anemia on the incidence of HAEC after pull-through.

Our results show that the incident of HAEC after TEPT was not associated either with age at HSCR diagnosis nor age at TEPT performed (*p* = 0.15 and 0.33, respectively, Table 3). Previous study also reported no significant increase in post-operative HAEC following pull-through with delayed diagnosis or timing of surgery (24, 25). Another study showed that HAEC admissions decreased by 30% with each doubling of age at diagnosis. Early initial management might be more important than definitive surgery itself, for example, colonic irrigation to reduce the size of distended colon and giving probiotics to maintain intestinal integrity, and controlling cytokines' regulatory effect, which would affect the maturation and development of gut immunity (26–28).

One study showed that patients with underweight status have higher proportion of colitis/enteritis after colorectal resection when compared to normal or obese patients (29). Although not statistically significant as a risk factor, malnutrition might contribute to HAEC incidence by its correlation with low hemoglobin and albumin level (29, 30).

We found that hypoalbuminemia during TEPT procedure did not correlate with HAEC incidence after TEPT procedure (Table 3). However, a previous study found that any decrease of albumin level from its normal level has serious outcomes for each 1 g/dl albumin level drop in colorectal surgical patients (31). This discrepancy might be due to different outcomes being measured, while we specifically looked for incident of HAEC in after TEPT procedure. Despite its insignificance as a prognostic factor for HAEC after TEPT, the level of albumin >3.5 g/dl before doing a surgery is important to be considered due to its relation to recovery time, length of stay, morbidity, and mortality in colorectal surgery (31).

Another novelty of our study is we evaluated maternal variables as possible risk factors of HAEC [vs. patient risk factors, i.e., sex, length of aganglionosis, and presence of other major anomalies (10, 11)]. Although, mothers with higher education level may have easier understanding about the acceptance of diagnostic measures, procedures, and risks, we did not find any difference in HAEC incidence between low and high education level (32).

Previous studies suggested that infants born preterm have looser tight junction, less goblet cell secretion, lesser Paneth cells number and secretory function, and lack of IgA secretion and this lack of immunity can lead to higher risk of developing HAEC (33). But, we also failed to prove the correlation and this insignificancy might be associated with the low number of preterm HAEC infants in this study, i.e., one case.

Infants born from mothers with age more than 35 years old have higher tendency to have trisomy 21, while trisomy 21 might increase the incidence of HAEC (34). It might be associated with the fact that trisomy 21 infants show less effective immune response and poorer wound healing (34). However, our study failed to show the association between mothers' age at childbirth and HAEC after TEPT.

Limitations of this study include its retrospective nature and data were subjected to different confounding variables such as variation of follow-up times and various surgeons performing TEPT. Since the medical records were used, our study might bias toward patients with post-operative HAEC and exclude patients with fewer HAEC criteria. Moreover, further prospective multicenter study is necessary and important to clarify and confirm our results.

## Conclusions

This report is the first study comparing two different cut-off values to evaluate the frequency of HAEC after TEPT and results suggest further using cut-off of ≥4 to expand the diagnosis of HAEC. Moreover, we also show for the first time that hemoglobin level is a strong risk factor for the HAEC development after TEPT.

## Data Availability Statement

The original contributions presented in the study are included in the article/supplementary material, further inquiries can be directed to the corresponding author/s.

## Ethics Statement

The studies involving human participants were reviewed and approved by the Medical and Health Research Ethics Committee of the Faculty of Medicine, Public Health and Nursing, Universitas Gadjah Mada/Dr. Sardjito Hospital. Written informed consent to participate in this study was provided by the participants' legal guardian/next of kin.

## Author Contributions

G, RL, BA, and FA conceived the study. RL, SK, BA, FA, RP, and G collected and analyzed the data. SK drafted the manuscript. GS, AR, GA, AD, AM, and G critically revised the manuscript for important intellectual content. G, AD, and AM facilitated all project-related tasks. All authors have read and approved the manuscript and agreed to be accountable for all aspects of the work in ensuring that questions related to the accuracy or integrity of any part of the work are appropriately investigated and resolved.

## Conflict of Interest

The authors declare that the research was conducted in the absence of any commercial or financial relationships that could be construed as a potential conflict of interest.

## Publisher's Note

All claims expressed in this article are solely those of the authors and do not necessarily represent those of their affiliated organizations, or those of the publisher, the editors and the reviewers. Any product that may be evaluated in this article, or claim that may be made by its manufacturer, is not guaranteed or endorsed by the publisher.
